# Prognostic Impact of CT-Quantified Muscle and Fat Distribution before and after First-Line-Chemotherapy in Lung Cancer Patients

**DOI:** 10.1371/journal.pone.0169136

**Published:** 2017-01-20

**Authors:** Johanna Nattenmüller, Raoul Wochner, Thomas Muley, Martin Steins, Simone Hummler, Birgit Teucher, Joachim Wiskemann, Hans-Ulrich Kauczor, Mark Oliver Wielpütz, Claus Peter Heussel

**Affiliations:** 1 Department of Diagnostic and Interventional Radiology, University Hospital, Im Neuenheimer Feld, Heidelberg, Germany; 2 Translational Lung Research Center Heidelberg (TLRC), Member of the German Center for Lung Research (DZL), Im Neuenheimer Feld, Heidelberg, Germany; 3 Department of Diagnostic and Interventional Radiology with Nuclear Medicine, Thoraxklinik at University of Heidelberg, Amalienstraße, Heidelberg, Germany; 4 Translational Research Unit (STF), Thoraxklinik at University of Heidelberg, Amalienstraße, Heidelberg, Germany; 5 Department of Thoracic Oncology, Thoraxklinik at University of Heidelberg, Amalienstraße, Heidelberg, Germany; 6 Department of Pneumology and Respiratory Critical Care Medicine, Thoraxklinik at University of Heidelberg, Amalienstraße, Heidelberg, German; 7 Division of Medical Oncology, National Center for Tumor Diseases (NCT); Heidelberg University Hospital, Im Neuenheimer Feld, Heidelberg, Germany; University of Nebraska Medical Center, UNITED STATES

## Abstract

**Introduction:**

Cachexia and sarcopenia are associated with poor outcome and increased chemotherapy-induced toxicity in lung cancer patients. However, the complex interplay of obesity, sarcopenia and cachexia, and its impact on survival in the context of first-line-chemotherapy is not yet understood.

**Methods:**

In 200 consecutively recruited lung cancer patients (70 female, mean age 62y; mean BMI 25 kg/m^2^; median follow-up 15.97 months) with routine staging-CT before and after chemotherapy (CTX, mean interval: 4.3 months), densitometric quantification of total (TFA), visceral (VFA), and subcutaneous-fat-area (SFA), inter-muscular-fat-area (IMFA), muscle-density (MD), muscle-area (MA) and skeletal-muscle-index (SMI) was performed retrospectively to evaluate changes under chemotherapy and the impact on survival.

**Results:**

We observed increases in TFA, VFA, SFA, VFA/SFA, and IMFA (p<0.05–0.001), while there were decreases in MA, MD and BMI (p<0.05–0.001) after chemotherapy. High pre-therapeutic VFA/SFA was a predictive factor for poor survival (HR = 1.272; p = 0.008), high pre-therapeutic MD for improved survival (HR = 0.93; p<0.05). Decrease in BMI (HR = 1.303; p<0.001), weight (HR = 1.067; p<0.001) and SMI (HR = 1.063; p<0.001) after chemotherapy were associated with poor survival. Patients with ≥4 CTX-cycles showed increased survival (17.6 vs. 9.1months), less muscle depletion (SMI_difference_: p<0.05) and no BMI loss (BMI_difference_: p<0.001).

**Conclusions:**

After chemotherapy, patients exhibited sarcopenia with decreased muscle and increased adipose tissue compartments, which was not adequately mirrored by BMI and weight loss but by imaging. Particularly sarcopenic patients received less CTX-cycles and had poorer survival. As loss of BMI, weight and muscle were associated with poor survival, early detection (via imaging) and prevention (via physical exercise and nutrition) of sarcopenia may potentially improve outcome and reduce chemotherapy-induced toxicity.

## Introduction

Lung cancer is one of the most common cancers with the highest incidence worldwide in men and the third highest incidence in women. In terms of mortality, it ranks highest in men and second highest in women [1, 2].

Cachexia is observed in many lung cancer patients and “is defined as a multifactorial syndrome characterized by an ongoing loss of skeletal muscle mass (with or without loss of fat mass) that cannot be fully reversed by conventional nutritional support and leads to progressive functional impairment” [3]. The most characteristic clinical sign of cachexia is unintended weight loss, which is caused by inflammatory processes, abnormal metabolism and reduced caloric intake [3, 4]. Cachexia is associated with reduced BMI, reduced physical capability and increased chemotherapeutic toxicity [3, 4]. More importantly, it is a predictor of poor outcome [4]. Sarcopenia, either on its own or as a central component of cachexia, is defined as muscle depletion “two standard deviations below the mean of a young reference group” [5]. In studies with cancer of the gastrointestinal-tract (GI), breast cancer and lung cancer, sarcopenia was associated with poor survival [4, 6–10]. Moreover, sarcopenia is associated with higher rates of chemotherapy-induced toxicity [11, 12].

On the other hand, the worldwide prevalence of overweight and obesity is still increasing and has already nearly doubled since 1980 [13, 14]. In addition to many known health burdens associated with obesity, such as the metabolic syndrome with an increased mortality risk [13, 15], the presence of cachexia can be masked in obese patients. This occurs especially in the case of sarcopenic obesity, which is defined as loss of muscle mass with a simultaneous increase of adipose tissue [16]. Patients who develop sarcopenic obesity show no or only little change in weight or BMI, and thus these processes, which are a symptom of cachexia, are hidden and can easily be missed. In obese patients with GI or respiratory tract cancer induced sarcopenia was an independent factor for poor survival [17].

As sarcopenia is an important component of cachexia with prognostic relevance and impact on the toxicity of chemotherapy, it is of great importance to measure sarcopenia instead of mere weight loss, especially when the widely spread obesity is potentially masking cachexia and sarcopenia. Thus, the aim of this study is to understand the interaction of sarcopenia, as central part of cachexia, and adipose tissue compartments over the course of chemotherapy in lung cancer patients.

We conducted a retrospective study, including 200 lung cancer patients in order to quantify the muscle and adipose tissue compartments before and after first-line-chemotherapy using densitometric CT-based quantification, which is an established method for muscle as well as adipose tissue characterization [4, 18]. We determined the changes in these parameters after chemotherapeutic treatment as well as baseline data before chemotherapy. The data were investigated for predictive parameters prior to chemotherapy as well as prognostic parameters with regard to changes after chemotherapy.

## Materials and Methods

This retrospective single center study was approved by the review board and the local ethics committee of the university hospital Heidelberg. All participants provided written informed consent.

### Patient demographics

200 lung cancer patients were consecutively recruited by alphabetically reviewing first-line-chemotherapy order lists of the hospital pharmacy for the years 2009 to 2012. Inclusion criteria were: histologically confirmed lung cancer, administration of first-line-chemotherapy, availability of two analyzable computed-tomography (CT) scans (one before and one after chemotherapy, coverage of vertebral-body L2/3), absence of soft-tissue metastases at level L2/3; availability of information about weight and height before and after chemotherapy, availability of survival data and no surgery in the time interval between CT-scans. Data on age, gender, date of initial diagnosis, histology of cancer, stage according to UICC and TMN, localization of potential metastases; duration, type and number of cycles of first-line-chemotherapy; potential surgery before chemotherapy; time of death or last contact with hospital or general practitioner; height, weight before and after chemotherapy; and technical details of CT-scans were retrieved from the hospital information system I.S.-H.*med. (SAP, Walldorf, Germany). For patients characteristics refer to Table 1.

**Table 1:** Characteristics of the study population, n = 200.

**Age (y),** mean ± SD	62.3 ± 9.5
**Sex,** male / female	130 (65%) / 70 (35%)
**BMI (kg/m^2^) baseline,** mean ± SD	24.9 ± 3.9
Underweight (< 18.5)	6 (3.0%)
Normal weight (18.5–24.9)	97 (48.5%)
Overweight (25.0–29.9)	79 (39.5%)
Obese Class I -III(≥ 30.0)	18 (9.0%)
**UICC Stage**	
Ia	0
Ib	3 (1.5%)
IIa	5 (2.5%)
IIb	5 (2.5%)
IIIa	16 (8.0%)
IIIb	27 (13.5%)
IV	144 (72.0%)
**Histology**	
**NSCLC**	175 (87.5%)
Adeno	121 (60.5%)
Squamous cell	39 (19.5%)
Large cell-neuroendocrine	4 (2.0%)
Other	11 (5.5%)
**SCLC**	25 (12.5%)
Chemotherapy*	
Carboplatin	183 (91.5%)
Cisplatin	57 (28.5%)
Gemcitabine	69 (34.5%)
Vinorelbine	48 (24.0%)
Etoposide	28 (14.0%)
Pemetrexed	33 (16.5%)
Other	44 (22.0%)
**Number of CTX cycles**	
1	3 (1.5%)
2	28 (14.0%)
3	17 (8.5%)
4	67 (33.5%)
5	11 (5.5%)
6	73 (36.5%)
8	1 (0.5%)
**Surgery before CTX**	52 (26.0%)
**Δt CT scans, months,** mean ± SD	4.4 ±1.6

*Multiple entries possible due to regimen change and combined protocols containing several agents.

### Quantification of adipose tissue compartments and muscle via CT

We retrospectively retrieved chest CT-scans from the institutional PACS (Synapse, Fuji, Düsseldorf, Germany). Contrast-enhanced CT-scans were performed with intra-individually identical exposure parameters before and after chemotherapy, and reconstructed with an overlapping slice thickness of 3mm.

#### Adipose tissue

Area-based quantification of adipose tissue compartments was performed on one image slice between vertebral-body L2/3 with a semiautomatic volume tool (Syngo Volume tool, Siemens Healthcare, Munich, Berlin, Germany). By manually determining specific regions of interest (ROI), the Total-Fat-Area (TFA, whole circumference abdomen) and the Visceral-Fat-Area (VFA, along the fascial plane tracing the abdominal wall) were measured (volumetric quantification of selected slice, divided by slice thickness) [19]. Adipose tissue within these ROIs was selected by limiting the measurement thresholds to a lower attenuation limit of -190HU and an upper attenuation limit of -30HU as previously described [19, 20]. Subcutaneous-fat-area (SFA) was calculated by subtracting VFA from TFA and the visceral to subcutaneous fat ratio was calculated as VFA/SFA [19].

#### Muscle

On the identical image slice (L2/3) a specific ROI covering all muscles (M. psoas major, M. erector spinae, M. quadratus lumborum, M. latissimus dorsi, M. transversus abdominis, M. obliquus internus abdominis and externus abdominis and M. rectus abdominis) was manually selected using the Syngo Volume Tool (volumetric quantification of selected slice, divided by slice thickness). The muscle tissues were first selected by limiting the measurements to a wide spectrum with a lower attenuation limit of -29HU and an upper attenuation limit of 150HU (MA_150_) as previously described [4, 21], secondly a limit of +40 to +100HU (MA_100_) was chosen to measure the more dense parts of muscle tissue (without fat infiltration) and lastly a limit of -190 to -30HU to measure only adipose tissue within muscle compartments (IMFA) [19]. The skeletal-muscle-index (SMI) was calculated as SMI = muscle_150_/(height^2^) with the unit cm^2^/m^2^. Mean muscle density in HU of each ROI was recorded (MD) [4, 17].

### Differences of parameters before & after chemotherapy

For all recorded tissue parameters (TFA, VFA, SFA, MA_150_, MA_100_, IMFA, MD_150,_ MD_100_, SMI, VFA/SFA, BMI, weight) the difference between time points of the CT-scan before (CT1) and after chemotherapy (CT2) were calculated as:

$$\Delta parameter=parameter_{CT1}–parameter_{CT2}$$

Thus, a positive value of parameter_diff_ shows a decrease and a negative value an increase of the respective parameter. A change of 0±2% was regarded as stable, >2% was categorized as decrease and <-2% as increase [22].

### Statistical analysis

Data were recorded using a spreadsheet program (Microsoft Office Excel® 2010, Microsoft Corporation, Redmond, WA, USA). Statistical analyses were performed using SPSS Statistics (Version 21, IBM, Armonk, NY, USA). Data were tested for normality using the Shapiro-Wilk-test. As some data were not normally distributed non-parametric tests were used subsequently. Group comparisons were performed using the Mann-Whitney-U test. The Wilcoxon-signed-rank test was used to test the parameters before and after chemotherapy. The impact of muscle and fat parameters, age, gender, BMI, weight and stage on overall survival was tested with the Kaplan-Meier-method and Cox regression, including calculations of the Hazard-Ratio (HR). Categorical variables included gender, histology (NSCLC vs. SCLC), stage, (I/II vs. III and vs. IV), surgery, and number of CTX-cycles (1–3 vs. ≥4). To analyze continuous variables using the Kaplan-Meier-method, data were transformed into dichotomic data using a threshold value, which was determined by the software tool ADAM (Version 2.54, Division of Biostatistics, DKFZ, Heidelberg, Germany). For comparison of two Kaplan-Meier-curves the Log-Rank-test was used. Results were regarded as statistically significant for p<0.05.

## Results

### Distribution of adipose tissue parameters before and after chemotherapy

As shown in Table 2, there was a small but significant decrease in BMI after chemotherapy (mean = 0.2kg/m^2^; p = 0.047), while the mean decrease in weight was not significant (mean = 0.5kg). All adipose tissue compartments increased significantly (TVA: mean = -17.3cm^2^, p = 0.001; VFA: mean = -9.1cm^2^, p = 0.001; SFA: mean = -5.9cm^2^, p = 0.04). There was also a significant increase in the ratio VFA/SFA (VFA/SFA_before_CTX_ = 1.4, VFA/SFA_after_CTX_ = 1.5. p = 0.006).

**Table 2:** Distribution (area in cm^2^) of adipose tissue across compartments quantified at level L2/3 using baseline CT-scans before (CT1) and follow-up CT after chemotherapy (CT2). BMI (kg/m^2^) and weight (kg) at time points of CT-scan before and after CTX. Wilcoxon-signed-rank-test for identifying significant differences of mean values before and after chemotherapy.

	TFA	VFA	SFA	VFA/SFA	BMI	Weight
**Baseline CT**						
**Mean**	292.3	149.6	115.7	1.4	24.9	72.5
**SD**	149.2	102.2	55.6	0.9	3.9	14.5
**Minimum**	35.8	11.2	10.8	0.1	16.4	43.0
**Maximum**	765.1	490.0	289.5	4.7	38.6	111.0
**Follow-up CT**						
**Mean**	309.6	158.7	121.6	1.5	24.8	72.0
**SD**	152.9	105.3	59.1	1.0	3.9	14.4
**Minimum**	33.1	10.2	6.3	0.2	16.4	42.0
**Maximum**	751.5	506.0	285.2	5.0	35.6	110.0
**Difference CT1-CT2**						
**Mean**	-17.3	-9.1	-5.9	-0.1	0.2	0.5
**p-value**	0.001*	0.001*	0.04*	0.006*	0.047*	0.06

SD = standard deviation. TFA = total fat area, VFA = visceral fat area, SFA = subcutaneous fat area, visceral fat ratio VFA/SFA and BMI = body-mass-index.

* = significant (p<0.05).

### Changes of muscle parameters before and after chemotherapy

There was a significant decrease of muscle mass, especially of the more dense muscle (muscle_100_) (muscle_150:_ mean = 4.1cm^2^, p<0.001; muscle_100_: mean = 6.2cm^2^, p<0.001) as well as SMI (mean = 1.4, p<0.001), while there was a significant increase of inter-muscular-fat-area (IMFA: mean = -2.0, p<0.001). The mean attenuation of muscle tissue was significantly decreased after chemotherapy (MD_150:_ mean = 2.1HU, p<0.001; MD_100_: mean = 0.8HU, p<0.001). See Table 3.

**Table 3:** Distribution (area in cm^2^) and mean attenuation (in HU) of muscle tissue compartments quantified at level L2/3 using CT-scans before (CT1) and after chemotherapy (CT2), Wilcoxon-signed-rank-test for identifying significant differences of mean values before and after chemotherapy.

	Muscle_150_	Muscle_100_	IMFA	SMI	MD_150_	MD_100t_
**Baseline CT**						
**Mean**	133.4	67.2	27.1	45.7	38.5	60.0
**SD**	31.8	22.3	12.9	8.7	7.3	2.8
**Minimum**	65.5	8.3	3.7	26.1	9.7	50.8
**Maximum**	215.6	138.8	76.5	66.5	59.6	66.1
**Follow-up CT**						
**Mean**	129.2	61.0	29.1	44.3	36.4	59.3
**SD**	31.3	20.8	13.3	8.6	7.6	3.0
**Minimum**	59.0	12.2	1.4	26.2	11.0	52.8
**Maximum**	220.4	114.6	73.2	67.7	55.0	66.4
**Difference CT1-CT2**						
**Mean**	4.1	6.2	-2.0	1.4	2.1	0.8
**p-value**	<0.001*	<0.001*	<0.001*	<0.001*	<0.001*	<0.001*

SD = standard deviation, IMFA = inter-muscular-fat-area, SMI = skeletal-muscle-index.

* = significant (p<0.05).

At time of baseline CT men and women showed different characteristics with regards to body composition (Table 4). Weight and BMI were significantly higher in men. Also, TFA and VFA were significantly higher in men. SFA was not significantly different. VFA/SFA was significantly higher in men, corresponding to a higher proportion of visceral than subcutaneous adipose tissue in men. Muscle area and SMI were also significantly higher in men.

**Table 4:** Distribution (area in cm2) of adipose tissue and muscle compartments among men and women quantified at level L2/3 using baseline CT-scans before (CT1) chemotherapy. BMI (kg/m2) and weight (kg) at baseline. Mann-Whitney-U-Test for identifying significant differences of mean values between men and women.

	Women	Men	Difference of means	p-Value
Baseline CT	n = 70	n = 130		
	Mean(SD)	Mean (SD)		
**Weight (kg)**	60.2 (9.3)	79.2 (12,3)	-19.0	<0,001*
**BMI (kg/m^2^)**	23.0 (3.3)	26.0 (3.9)	-3.0	<0.001*
**TFA (cm^2^)**	211.3 (106.8)	336.0 (150.8)	-124.7	<0.001*
**VFA (cm^2^)**	72.1 (54.6)	191.3 (97.6)	-119.1	<0.001*
**SFA (cm^2^)**	118.6 (54.1)	114.1 (56.5)	4.5	0.4
**Muscle**_**150**_ **(cm^2^)**	103.1 (20.9)	149.7 (23.7)	-46.6	<0.001*
**Muscle**_**100**_ **(cm^2^)**	55.0 (18.3)	73.8 (21.6)	-18.7	<0.001*
**IMFA (cm^2^)**	20.6 (10.5)	30.6 (12.8)	-10.1	<0.001*
**MD**_150_ **(HU)**	39.3 (8.1)	38.1 (6.7)	1.3	0.042*
**MD**_**100**_ **(HU)**	60.1 (2.8)	60.0 (2.8)	0.04	0.847
**SMI (cm^2^/m^2^)**	39.3 (6.8)	49.1 (7.6)	-9.8	<0.001*
**VFA/SFA**	0.6 (0.5)	1.8 (0.8)	-1.2	<0.0001*

SD = standard deviation. TFA = total fat area, VFA = visceral fat area, SFA = subcutaneous fat area, Visceral fat ratio VFA/SFA, BMI = body-mass-index, IMFA = inter-muscular-fat-area and SMI = skeletal-muscle-index.

* = significant (p<0.05).

After chemotherapy there was a significantly higher decrease in muscle tissue among men than women (muscle_150_: mean_difference_ = -4.7; p = 0.022; muscle_100_: mean_difference_ = -4.7, p = 0.019 and SMI: mean_difference_ = -1.6, p = 0.035). Regarding the other parameters, there was no significant difference between men and women (Table 5).

**Table 5:** Mann-Whitney-U-Test for identifying significant differences of mean values between men and women (area in cm^2^) between baseline and follow-up CT-scans regarding adipose tissue and muscle compartments quantified at level L2/3, as well as BMI (kg/m^2^) and weight (kg).

	Women	Men	Difference of means	p-Value
Differences between baseline and follow-up CT	n = 70	n = 130		
	Mean(SD)	Mean (SD)		
**Weight (kg)**	-0.4 (3.7)	1.0 (5.12)	-1.4	0.055
**BMI (kg/m^2^)**	-0.1 (1.4)	0.4 (1.66)	-0.5	0.065
**TFA (cm^2^)**	-21.0 (51.1)	-15.2 (76.36)	-5.8	0.68
**VFA (cm^2^)**	-9.2 (21.4)	-9.1 (50.44)	-0.2	0.953
**SFA (cm^2^)**	-9.5 (32.6)	-4.0 (26.01)	-5.4	0.367
**Muscle**_**150**_ **(cm^2^)**	1.1 (7.9)	5.8 (14.35)	-4.7	0.022*
**Muscle**_**100**_ **(cm^2^)**	3.2 (6.0)	7.9 (12.86)	-4.7	0.019*
**IMFA (cm^2^)**	-2.3 (4.1)	-1.8 (8.51)	-0.5	0.625
**MD**_**150**_ **(HU)**	1.5 (3.2)	2.4 (4.11)	-0.9	0.177
**MD**_**100**_ **(HU)**	0.5 (2.1)	0.9 (2.09)	-0.3	0.211
**SMI (cm^2^/m^2^)**	0.4 (3.1)	1.9 (4.75)	-1.6	0.035*
**VFA/SFA**	-0.05 (0.1)	-0.09 (0.41)	0.04	0.755

SD = standard deviation. TFA = total fat area, VFA = visceral fat area, SFA = subcutaneous fat area, Visceral fat ratio VFA/SFA, BMI = body-mass-index, IMFA = inter-muscular-fat-area and SMI = skeletal-muscle-index.

* = significant (p<0.05).

At baseline patients older than 63 years had significantly higher BMI, higher adipose tissue compartments and higher VFA/SFA. The mean attenuation of muscle tissue was lower and inter-muscular-fat was significantly higher in the elderly, while the area of dense muscle_100_ was significantly lower (Table 6).

**Table 6:** Distribution (area in cm^2^) of adipose tissue and muscle compartments quantified at level L2/3 using CT-scans before (CT1) chemotherapy in patients <63 years and ≥ 63 years. BMI (kg/m^2^) and weight (kg) at baseline. Mann-Whitney-U-Test for identifying significant differences of mean values between patients <63 years and ≥ 63 years.

Baseline CT	Patients	Patients	Difference of Means	p-value
	**< 63 years**	**≥ 63 years**		
	n = 98	n = 102		
	Mean (SD)	Mean(SD)		
**Weight (kg)**	70.8 (14.6)	74.2 (14.3)	-3.4	0.079
**BMI (kg/m^2^)**	24.1 (3.7)	25.8 (4.0)	-1.7	0.003*
**TFA (cm^2^)**	249.4 (134.9)	333.6 (151.2)	-84.1	<0.001*
**VFA (cm^2^)**	120.0 (84.1)	178.0 (110.1)	-58.0	<0.001*
**SFA (cm^2^)**	107.5 (57.9)	123.6 (52.4)	-16.1	0.016*
**Muscle**_**150**_ **(cm^2^)**	132.9 (33.5)	133.8 (30.3)	-0.9	0.735
**Muscle**_**100**_ **(cm^2^)**	74.3 (23.4)	60.4 (19.1)	13.8	<0.001*
**IMFA (cm^2^)**	22.0 (10.6)	32.0 (13.1)	-10.1	<0.001*
**MD**_**150**_ **(HU)**	42.2 (6.1)	35.0 (6.6)	7.1	<0.001*
**MD**_**100**_ **(HU)**	60.8 (2.8)	59.3 (2.6)	1.4	<0.001*
**SMI (cm^2^/m^2^)**	45.1 (8.9)	46.3 (8.5)	-1.2	0.179
**VFA/SFA**	1.2 (0.9)	1.5 (0.9)	-0.3	0.006*

SD = standard deviation. TFA = total fat area, VFA = visceral fat area, SFA = subcutaneous fat area, Visceral fat ratio VFA/SFA, BMI = body-mass-index, IMFA = inter-muscular-fat-area and SMI = skeletal-muscle-index.

* = significant (p<0.05).

After chemotherapy in patients younger than 63 years, there was a significantly higher increase in adipose tissue compartments (TFA: mean_difference_ = -22.1cm^2^ p = 0.035; VFA: mean_difference_ = -11.6cm^2^, p = 0.031; SFA: mean_difference_ = -9.9cm^2^, p = 0.007). All other parameters showed similar changes in both age groups (Table 7).

**Table 7:** Mann-Whitney-U-Test for identifying significant differences of mean values between patients <63 years and ≥ 63 years (area in cm^2^) between baseline and follow-up CT-scans regarding adipose tissue and muscle compartments quantified at level L2/3, as well as BMI (kg/m^2^) and weight (kg).

Differences between baseline and follow-up CT	Patients	Patients	Difference of Means	p-value
	< 63 years	≥ 63 years		
	Mean	Mean		
	(SD)	(SD)		
**Weight (kg)**	0.3 (5.2)	0.8 (4.1)	-0.5	0.811
**BMI (kg/m^2^)**	0.1 (1.7)	0.3 (1.4)	-0.1	0.835
**TFA (cm^2^)**	-28.5 (70.8)	-6.4 (64.8)	-22.1	0.035*
**VFA (cm^2^)**	-15.1 (39.4)	-3.4 (44.7)	-11.6	0.031*
**SFA (cm^2^)**	-11.0 (32.4)	-1.1 (23.4)	-9.9	0.007*
**Muskel**_**150**_ **(cm^2^)**	3.9 (12.0)	4.4 (13.3)	-0.5	0.776
**Muskel**_**100**_ **(cm^2^)**	6.5 (11.6)	6.0 (10.7)	0.5	0.654
**IMFA (cm^2^)**	-2.5 (5.9)	-1.5 (8.4)	-1.0	0.400
**Muskel**_**150**_ **(HU)**	2.1 (3.9)	2.1 (3.8)	-0.1	0.919
**Muskel**_**100**_ **(HU)**	0.7 (2.3)	0.9 (1.9)	-0.2	0.475
**SMI (cm^2^/m^2^)**	1.3 (4.0)	1.5 (4.5)	-0.2	0.712
**VFA/SFA**	-0.07 (0.3)	-0.08 (0.4)	0.01	0.701

SD = standard deviation. TFA = total fat area, VFA = visceral fat area, SFA = subcutaneous fat area, Visceral fat ratio VFA/SFA, BMI = body-mass-index, IMFA = inter-muscular-fat-area and SMI = skeletal-muscle-index.

* = significant (p<0.05).

The big subgroup of patients without prior surgery (n = 148) (see Tables 8 and 9) shows a similar change of muscle and adipose tissue compared to the total patient collective (Tables 2 and 3) distribution before and after chemotherapy with a significant decrease of muscle tissue and muscle attenuation with a simultaneous increase of adipose tissue and inter-muscular fat. While there was a small but significant decrease of BMI in the total patient collective (Table 2), BMI was not significantly decreased in the subgroup without prior surgery (Table 8). In both groups there was no significant change of weight.

**Table 8:** Distribution (area in cm^2^) of adipose tissue across compartments quantified at level L2/3 using baseline CT-scans before (CT1) and follow-up CT after chemotherapy (CT2) in subgroup of patients without surgery. BMI (kg/m^2^) and weight (kg) at time points of CT-scan before and after CTX. Wilcoxon-signed-rank-test for identifying significant differences of mean values before and after chemotherapy.

	TFA	VFA	SFA	VFA/SFA	BMI	Weight
**Baseline CT**						
**Mean**	290.3	149.6	113.5	1.4	25.0	72.4
**SD**	151.9	103.6	54.2	0.9	4.0	15.1
**Minimum**	35.8	11.2	10.8	0.1	16.4	43.0
**Maximum**	765.1	490.0	284.9	4.7	35.8	111.0
**Follow-up CT**						
**Mean**	308.4	159.5	119.2	1.5	24.7	71.8
**SD**	159.6	108.5	57.9	0.9	4.0	15.4
**Minimum**	33.1	10.2	6.27	0.2	16.4	42.0
**Maximum**	751.5	485.7	285.2	5.0	35.2	110.0
**Difference CT1-C2**	** **	** **	** **	** **	** **	
**Mean**	-18.1	-9.9	-5.7	-0.1	0.3	0.6
**p-value**	0.002*	0.003*	0.045*	0.036*	0.059	0.083

SD = standard deviation. TFA = total fat area, VFA = visceral fat area, SFA = subcutaneous fat area, visceral fat ratio VFA/SFA and BMI = body-mass-index.

* = significant (p<0.05).

**Table 9:** Distribution (area in cm^2^) and mean attenuation (in HU) of muscle tissue compartments quantified at level L2/3 using CT-scans before (CT1) and after chemotherapy (CT2) in subgroup of patients without surgery, Wilcoxon-signed-rank-test for identifying significant differences of mean values before and after chemotherapy.

	Muscle_150_	Muscle_100_	IMFA	SMI	MD_150_	MD_100t_
**Baseline CT**						
**Mean**	132.2	66.1	27.3	45.4	38.2	59.9
**SD**	32.2	22.8	13.4	8.5	7.5	2.9
**Minimum**	65.5	8.3	3.7	26.1	9.7	50.8
**Maximum**	215.6	138.8	76.5	65.8	59.6	66.1
**Follow-up CT**						
**Mean**	128.0	59.4	29.4	44.0	36.0	59.3
**SD**	32.5	20.9	14.0	8.7	7.7	2.9
**Minimum**	59.0	12.2	1.4	26.2	16.6	52.8
**Maximum**	220.4	114.6	68.2	67.7	55.0	65.8
**Difference CT1-2**	** **	** **	** **	** **	** **	** **
**Mean**	4.2	6.7	-2.1	1.4	2.2	0.6
**p-value**	<0.001*	<0.001*	<0.001*	<0.001*	<0.001*	0.001*

SD = standard deviation, IMFA = inter-muscular-fat-area, SMI = skeletal-muscle-index.

* = significant (p<0.05).

### Survival analysis

**Fig 1:**
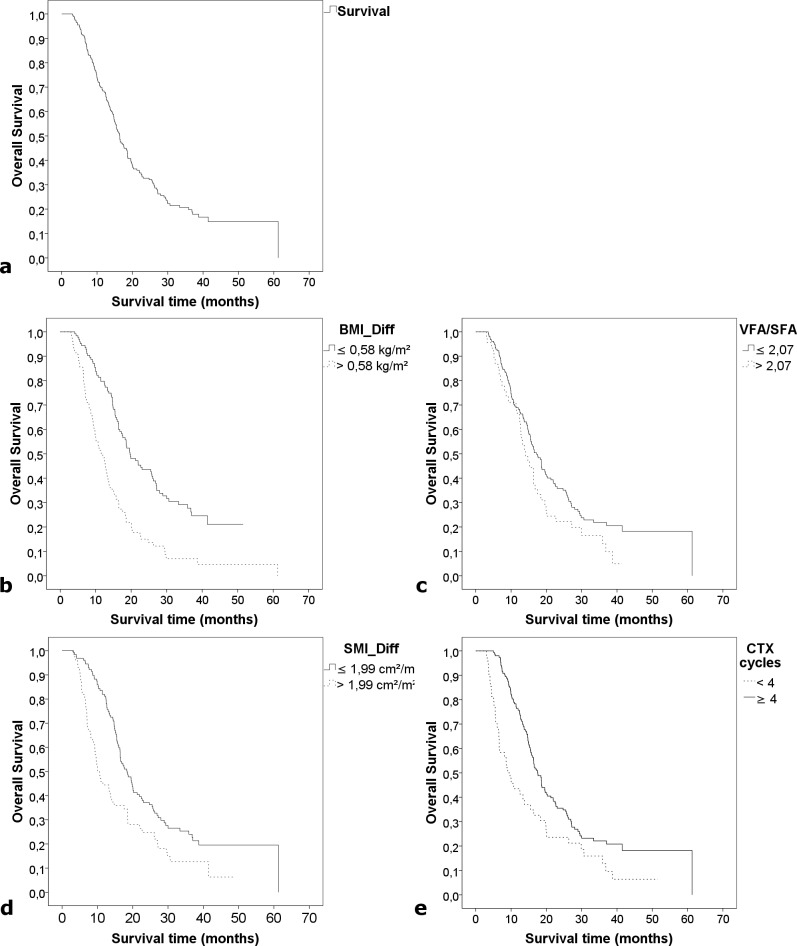
a) Overall survival with Kaplan-Meier method, n = 200. b) Median survival (11.6 months) of patient group with BMI difference ≥ 0.58kg/m^2^ (n = 76) vs. median survival (19.7 months) of patient group with BMI difference < 0.58 kg/m^2^ (n = 124) with Kaplan-Meier method, (p<0.001). c) Median survival (14.1 months) of patient group (n = 45) with VFA/SFA at baseline > 2.07 vs. median survival (17.2 months) of patient group (n = 155) with VFA/SFA at baseline ≤2.07; with Kaplan-Meier method. d) Median survival (10.1 months) of patient group (n = 72) with SMI difference > 1.99 cm^2^/m^2^ vs. median survival (18.4 months) of patient group (n = 128) with SMI difference ≤ 1.99 cm^2^/m^2^, with Kaplan-Meier method, p<0.001. e) Median survival (9.1 months) of patient group (n = 48) with<4 CTX cycles vs. median survival (17.6 months) of patient group (n = 152) with ≥4 cycles, with Kaplan-Meier method, p<0.001.

Mean patient follow-up was 18.9 months. The median overall survival was 16.4 months (95% CI 14.7–18.1 months) with a minimum of 3.0 and a maximum of 61.3 months (Fig 1A). At the time point of last data analysis 155 patients (77.5%) had deceased, 45 (22.5%) were still alive.

Among the clinical parameters, male gender, SCLC, higher tumor stages, no surgery and a lower number of CTX-cycles were associated with lower survival, see Table 10.

**Table 10:** Univariate survival analysis of clinical parameters with calculation of Hazard-Ratios (HR) using Cox-Regression, n = 200.

	Hazard-Ratio	KI_95%-Lower HR	KI_95%-Higher HR	p-value
Clinical parameters				
**Gender**	1.417	1.008	1.993	0.045*
**Histology: SCLC vs. NSCLC**	0.574	0.366	0.899	0.015*
**Stage: (I+II) vs. III**	1.663	0.722	3.834	0.232
**Stage (I+II) vs. IV**	2.982	1.375	6.471	0.006*
**Stage III vs. IV**	0.558	0.369	0.843	0.006*
**Age**	1.007	0.989	1.025	0.444
**Surgery: yes vs.no**	0.632	0.432	0.923	0.018*
**Number of CTX cycles**	0.556	0.390	0.792	0.001*

* = significant (p<0.05).

At baseline, BMI had no influence on survival. Higher VFA/SFA was significantly associated with lower survival (HR = 1.272; p = 0.008), which was also shown using the Kaplan-Meier-method (Fig 1C). High muscle tissue attenuation was associated with better survival (MD_100_: HR = 0.93, p = 0.011) (Table 11). Muscle-area and adipose tissue had no significant impact on survival (non-significant data not shown).

**Table 11:** Univariate survival analysis of BMI, adipose tissue and muscle parameters at baseline with calculation of Hazard-Ratios (HR) using Cox-Regression, n = 200; * = significant (p<0.05).

	Hazard-Ratio	KI_95%-Lower HR	KI_95%-Higher HR	p-value
Baseline CT				
**BMI**	1.019	0.978	1.061	0.365
**MD**_**100**_	0.93	0.879	0.984	0.011*
**VFA/SFA**	1.272	1.064	1.522	0.008*

Visceral fat ratio VFA/SFA, MD = muscle density, BMI = body-mass-index.

* = significant (p<0.05).

After chemotherapy a decrease of weight (HR = 1.067) and BMI (HR = 1.303) was significantly associated with lower survival (each p = <0.001), which was also shown using the Kaplan-Meier-method (Fig 1B). Loss of muscle tissue (SMI and muscle_150_) was associated with a risk for lower survival with a Hazard Ratio_SMI_ of 1.063 (p_SMI_ = 0.000721), which was also shown using the Kaplan-Meier method (Fig 1D). Regarding adipose tissue compartments, only an increase in SFA was significantly associated with lower survival (HR = 1.006, P = 0.046) (Table 12). Muscle density and adipose tissue differences had no significant impact on survival (non-significant data not shown).

**Table 12:** Univariate survival analysis of differences of BMI, adipose tissue and muscle parameters between baseline and follow-up CT-scans after chemotherapy (CT1-CT2) with calculation of Hazard-Ratios (HR) using Cox-Regression, n = 200.

Differences between baseline and follow-up CT	Hazard-Ratio	KI_95%-Lower HR	KI_95%-Higher HR	p-value
**Weightloss (%)**	1.067	1.040	1.095	<0.001*
**BMI**	1.303	1.171	1.451	<0.001*
**VFA**	1.003	0.999	1.007	0.185
**SFA**	1.006	1.000	1.012	0.046*
**SMI**	1.063	1.026	1.101	0.000721*
**Muscle**_**150**_	1.021	1.009	1.034	0.000884*
**VFA/SFA**	0.745	0.436	1.271	0.280

Visceral fat ratio VFA/SFA, MD = muscle density, BMI = body-mass-index, VFA = visceral fat area, SFA = subcutaneous fat area and SMI = skeletal-muscle-index.

* = significant (p<0.05).

### Correlation of muscle, VFA/SFA and BMI with number of chemotherapy cycles

As the number of CTX-cycles had a strong impact on survival (17.6 months in patients with ≥4cycles vs. 9.1 months in patients with 1–3 months, p = 0.001, Fig 1E), the relationship of this number to VFA/SFA at baseline and to changes in SMI_150_ and BMI after chemotherapy, all of which also had impact on survival, was analyzed. Patients who received 1–3 CTX-cycles showed significantly higher loss of muscle tissue (SMI_150_: mean_difference_ = 1.3, p = 0.013). BMI also decreased more in patients with 1–3 cycles than in patients with ≥4 cycles, who had nearly stable BMI (mean_difference_ = 0.95, p = 0.000136). There was no significant difference regarding VFA/SFA in both groups (Table 13).

**Table 13:** Differences of SMI, VFA/SFA and BMI (CT1-CT2) tested with Mann Whitney-U-Test in patients with 1–3 CTX-cycles (n = 48) vs. ≥4 CTX-cycles (n = 152).

	<4 cycles	≥4 cycles	Difference	p-value
**SMI**_**150-difference**_ **(cm^2^/m^2^)**				
**Mean (SD)**	2.4 (3.9)	1.1 (4.4)	1.3	0.013*
**VFA/SFA**_**difference**_				
**Mean (SD)**	1.6 (1.0)	1.3 (0.8)	0.3	0.107
**BMI**_**difference**_ **(kg/m^2^)**				
**Mean (SD)**	0.9 (1.3)	-0.04 (1.6)	0.95	0.000136*

SD = standard deviation. Visceral fat ratio VFA/SFA, BMI = body-mass-index and SMI = skeletal-muscle-index.

* = significant (p<0.05).

## Discussion

This retrospective study including 200 lung cancer patients aims to understand the complex interaction of sarcopenia as part of cachexia with adipose tissue during the course of first-line-chemotherapy. We first evaluated whether there was a change in muscle and adipose tissue compartments after first-line-chemotherapy by quantifying CT-scans before and after CTX. Second, as sarcopenia and cachexia are of great prognostic relevance we investigated whether muscle or adipose tissue parameters at baseline or their change after chemotherapy had prognostic impact on patient survival. This is the first study to combine an evaluation of the distribution and change of both adipose and muscle tissue including inter-muscular adipose tissue in the setting of first-line-chemotherapy in lung cancer patients analyzing CT-data at two time points before and after chemotherapy.

Our results showed that both adipose and muscle tissue compartments significantly changed during chemotherapy within a relatively short period of time (mean time interval 4.4months): all adipose tissue compartments (TFA, VFA and SFA) increased, especially in favor of visceral adipose tissue with an increasing ratio of VFA/SFA. The muscle tissue, especially the more dense muscle tissue, and the muscle attenuation decreased, while the fat fraction within the muscle compartment increased. These changes were not mirrored by any significant change in weight and BMI decreased by a small amount only.

Overall survival of the 200 patients was 16.4 months with a mean follow-up time of 18.9 months. Clinical parameters associated with lower survival were male gender, SCLC, higher tumor stages, no surgery and a lower number of CTX-cycles. Regarding muscle or adipose tissue parameters with impact on survival, we found a high ratio of VFA/SFA at baseline to be a significant predictor for lower survival. A high density of muscle tissue at baseline was a predictor for better survival. After chemotherapy a high loss of weight, BMI, muscle tissue (SMI and Muscle_150_) and increase of SFA were associated with poor survival.

Our patient population is representative for lung cancer patients regarding the distribution of gender, age as well as the different types of histology [23, 24]. Regarding BMI distribution at baseline mean BMI was nearly above normal weight with 24.9: 48.5% of patients had normal weight, while another 48.5% were overweight or obese, and only 3% were underweight. Regarding the distribution of adipose and muscle tissue at baseline we found significant differences between both gender and age groups, which were on par with results of previous studies: in men we found higher total and visceral adipose tissue, as well as a higher ratio of VFA/SFA, while subcutaneous adipose tissue was comparable between both genders [19]. Muscle tissue was significantly higher in men. With regard to age, older patients had higher BMI and adipose tissue compartments, while muscle area and attenuation were lower [5, 19].

After chemotherapy, men had a significantly higher decrease in muscle tissue than women, which was also seen by Kimura et al. [7]. Regarding age, all adipose tissue compartments increased significantly more in younger than in older patients. One reason for these differences in men and younger patients might be that these groups started from a higher baseline amount of muscle, and that muscle loss in the context of sarcopenia follows a hyperbolic pattern (i.e. the rate of change decreases with the amount of muscle tissue).

In literature, cachexia and weight loss are seen in many cancer patients, including lung cancer patients depending on the time point of observation during the course of the disease [3, 7, 25, 26]. In our collective 39% of all patients showed weight loss (mean across all patients was 0.54 kg but not statistically significant), while there was a small but significant loss of BMI.

As a component of cachexia, sarcopenia is also frequent in lung cancer patients: Prado et al. described sarcopenia in patient groups with lung and GI-tract cancer in about 15% and Baracos et al. in lung cancer patients with 46.8% [17, 27].

In our population, more patients showed signs of sarcopenia than weight loss or BMI change. Within a relatively short period of time from before until after chemotherapy (mean time interval 4.4 months) they exhibited a reduction of muscle area (in 49.5% of all patients) and attenuation (corresponding to a diffuse small-scale-fatty infiltration of muscle, which is not measurable within the limits of -190 to -30HU due to partial volume effect), and an increase of inter-muscular-fat-area (corresponding to a grander-scale-fatty infiltration, measurable within the limits of -190 to -30HU). Simultaneously we found an increase of all adipose tissue compartments (in 59.5% of patients (VFA)) in line with sarcopenic obesity in these patients. Changes in weight and BMI thus did not adequately reflect this strong ongoing loss of muscle tissue at that point of time within the course of disease in a large number of patients, as highlighted by the discrepancy in the number of patients with a loss of BMI/weight (39%) vs. loss of muscle area (49.5%) and increase in adipose tissue area (59.9%). This shows the importance of imaging techniques, which provide additional information beyond weight and BMI and are able to show occult muscle depletion, especially when there is a simultaneous increase in adipose tissue, as highlighted by other authors [17]. As sarcopenia and cachexia are of prognostic relevance, it is important not to miss muscle depletion and accompanying changes in body composition, which are not adequately reflected by weight and BMI changes because of conversion of muscle tissue to adipose tissue or because of the pathological presence of edema, ascites or pleural effusions.

Regarding survival, one major prognostic factor was the number of CTX-cycles. Patients who received more cycles lived significantly longer and showed less muscle depletion (1.1 vs. 2.4cm^2^/m^2^; p = 0.013), lower VFA/SFA (1.3 vs. 1.6; p = 0.107) and a small amount of BMI gain (-0.04 vs. 0.9kg/m^2^; p<0.001). This might be explained by CTX-induced tumor control with consecutively less cachexia, or because patients with preexisting cachexia/sarcopenia tolerated chemotherapy less and thus received fewer cycles and had worse tumor control resulting in lower survival. A reason for higher CTX-toxicity in patients with sarcopenia might be an unfavorable pharmacokinetic effect due to the reduced distribution volume within the body as a consequence of muscle depletion [11].

A high VFA/SFA ratio at baseline was associated with a poor prognosis. This was described by Rickles et al. in colorectal cancer patients and by George et al. in breast cancer patients with an increased all-cause mortality [28, 29]. However, visceral obesity in that study was determined by waist circumference and waist-to-hip ratio and not based on CT data [28, 29]. BMI at baseline had no prognostic effect in our patient group.

A high attenuation of muscle tissue at baseline, reflecting functionally intact muscle tissue without relevant fatty infiltration, was a predictor for better survival, which corresponds to results of Martin et al., where a low attenuation of muscle was a predictor for poor survival [4]. Also Sjøblom et al. showed in 734 patients with NSCLC before first line chemotherapy that high muscle attenuation was a positive prognostic factor in a multivariate analysis (HR = 0.98, p = 0.001) [30]. However, contradicting to our and other results, SMI had no significant impact on survival in their study [4, 7, 8, 30]. In contrast to our study Sjøblom et al analyzed one time point before chemotherapy and not a second time point to show changes after chemotherapy as we did in this study [30].

After chemotherapy loss of muscle tissue/mass (SMI and Muscle_150_) was a predictor for poor prognosis. The prognostic impact of this measure of sarcopenia corresponds to the results of several other studies: Kimura et al. showed in a study with 134 cases of NSCLC, that patients with cachexia and a low muscle index had a poorer prognosis [7]. Martin et al. demonstrated in a study with 1473 patients with GI or lung cancer that survival was poorest in patients with weight loss, sarcopenia and low muscle attenuation, regardless of weight. [4] Also Stene et al. observed in a study with 35 NSCLC-patients sarcopenia in 55% of patients, who did not respond to chemotherapy while 14 patients without weight loss exhibited a good response [31]. Thus muscle change was a significant prognostic factor of survival [31]. Kim et al. showed in 149 SCLC-patients by using two cutoff values (international and Korean cutoff) of SMI_L3_ that patients with sarcopenia had poorer survival (8.6 months versus 16.8 months; p = 0.031) [8]. However, Kim et al. as well as Martin et al. used CT-data at one time point after first cancer diagnosis [4, 8]. We investigated the development of sarcopenia after first-line-chemotherapy using CT-data of two time points (one before and one after chemotherapy). With this information we could directly show changes within this period of time, i.e. during first-line-chemotherapy. Beyond the muscle index we also investigated fat content within the muscle and abdominal adipose tissue and could show that abdominal and intramuscular adipose tissue increased while muscle tissue was decreased during chemotherapy. Kim et al. wrote in their methods part that they also measured adipose tissue, but they did not further described this in their results [8]. Also Kim et al. did not focus especially on patients with first-line chemotherapy [8].

Also, loss of BMI and weight after administration of chemotherapy was a predictor for poor prognosis, which is on par with earlier studies [4, 7].

Because of the prognostic relevance of sarcopenia, patients might profit if pre-existing imaging data (staging CT-scans performed for clinical reasons) could be re-evaluated to detect sarcopenia earlier than by changes in clinical parameters (e.g. weight or BMI), which occur at a later and potentially irreversible stage. Information gained from the analysis of imaging data could be used to adapt patients’ nutrition and physical exercise plans, as there is evidence that sarcopenia might be reversible in its early stages [3, 32, 33]. Furthermore, as sarcopenia is associated with higher CTX-toxicity, dose adjustments could be done in patients at risk [11, 17].

This study is subject to the following limitations. Because of its retrospective nature, missing data could not be analyzed, such as smoking status, nutritional habits or physical exercise. Ethnicity was not regularly recorded in patients’ files. However, the patient collective was presumably in large part Caucasian, as patients at the study site are mainly Caucasian. However, exceptions are possible as the ethnicity was not specially recorded.

Because routine staging CT-scans were used which implied i.v. contrast in a majority of cases, alterations of muscle perfusion may have influenced muscle attenuation. However, in nearly all cases the identical CT-protocol was used at all time-points which should minimize its influence. Our study population included patients with different types of histology (SCLC and NSCLC), but with a representative distribution. Patients with and without prior surgery were included, but not with surgery between both CT-scans. However, the big subgroup of patients without prior surgery showed a similar change of muscle and adipose tissue compared with the total patient collective (Table 5). The only exception was BMI, which showed no significant change anymore, which points to the fact that imaging methods are able to show sarcopenia earlier than BMI or weight change.

The median survival of our patient cohort was relatively long despite the fact that 72.0% of our patients were stage IV at baseline. Importantly, very obese patients with high SFA were not included if body parts were partly outside of the field-of-view (limit 500 mm). A systematic error cannot be ruled out as this is a single center trial with patients of a university hospital setting [18].

Summing up, sarcopenia as a central component of cachexia is an important prognostic factor associated with poor survival and increased toxicity of chemotherapy. Within a mean time interval of 4.4 months after chemotherapy, muscle tissue and density was decreased, while adipose tissue and inter-muscular adipose tissue were increased, reflecting sarcopenia and in particular sarcopenic obesity in our patient collective, which was only detectable by imaging. Sarcopenic patients received less CTX-cycles and had poorer survival, probably as consequence of elevated CTX-toxicity. As loss of BMI, weight and muscle mass were associated with poor survival, sarcopenia prevention, e.g. via physical exercise (resistance training) and nutritional intervention, may potentially improve outcome after chemotherapy.
